# A Self-Aligned a-IGZO Thin-Film Transistor Using a New Two-Photo-Mask Process with a Continuous Etching Scheme

**DOI:** 10.3390/ma7085761

**Published:** 2014-08-11

**Authors:** Ching-Lin Fan, Ming-Chi Shang, Bo-Jyun Li, Yu-Zuo Lin, Shea-Jue Wang, Win-Der Lee

**Affiliations:** 1 Graduate Institute of Electro-Optical Engineering, National Taiwan University of Science and Technology, Taipei City 106, Taiwan; E-Mails: d10019004@mail.ntust.edu.tw (M.-C.S.); m10019004@mail.ntust.edu.tw (B.-J.L.); 2 Department of Electronic Engineering, National Taiwan University of Science and Technology, Taipei City 106, Taiwan; E-Mail: D9802312@mail.ntust.edu.tw; 3 Institute of Materials Science and Engineering, National Taipei University of Technology, Taipei City 106, Taiwan; E-Mail: sjwang@ntut.edu.tw; 4 Department of Electrical Engineering, Lee-Ming Institute of Technology, New Taipei City 243, Taiwan; E-Mail: leewd@mail.lit.edu.tw

**Keywords:** amorphous indium–gallium–zinc–oxide (a-IGZO), back-side exposure, self-aligned process, thin-film transistor (TFT), two-photo-mask process, backside-lift-off (BLO)

## Abstract

Minimizing the parasitic capacitance and the number of photo-masks can improve operational speed and reduce fabrication costs. Therefore, in this study, a new two-photo-mask process is proposed that exhibits a self-aligned structure without an etching-stop layer. Combining the backside-ultraviolet (BUV) exposure and backside-lift-off (BLO) schemes can not only prevent the damage when etching the source/drain (S/D) electrodes but also reduce the number of photo-masks required during fabrication and minimize the parasitic capacitance with the decreasing of gate overlap length at same time. Compared with traditional fabrication processes, the proposed process yields that thin-film transistors (TFTs) exhibit comparable field-effect mobility (9.5 cm^2^/V·s), threshold voltage (3.39 V), and subthreshold swing (0.3 V/decade). The delay time of an inverter fabricated using the proposed process was considerably decreased.

## 1. Introduction

Numerous recent studies have focused on oxide semiconductors, such as amorphous indium–gallium–zinc oxide (a-IGZO). Because of their high mobility and transparency, these semiconductors have been applied as active channel layers in thin-film transistors (TFTs) [1,2,3]. Regarding traditional silicon-based TFTs, amorphous silicon (a-Si:H) exhibits low carrier mobility (0.5–1 cm^2^/V·s), whereas polycrystalline silicon (poly-Si) requires high-temperature fabrication processes (>500 °C) [4,5]. Conversely, a-IGZO TFTs can be fabricated on plastic substrates at low temperatures and exhibit excellent electrical characteristics [6,7].

The a-IGZO TFTs that are employed in displays are typically fabricated using back-channel-etching structure and five photomasks, including the definition of an etching-stop (ES) layer to protect the a-IGZO active layer from damage caused by etching the source/drain (S/D) electrodes [8]. However, thin-film transistors (TFTs) that involve an ES require a misalignment margin for the ES to ensure the good contact between the S/D and the induced channel; thus, high parasitic capacitances which between the source/drain (S/D) electrodes and gate electrode (*C*_gd_, *C*_gs_) could occur, decreasing the operational speed of the TFT circuit [9].

To reduce the parasitic capacitance of TFTs with ES, Geng *et al.* proposed a self-aligned process employing backside-ultraviolet (BUV) exposure through a metal-gate-electrode to define the ES area, thereby reducing the misalignment margin [10]. However, four or five-photo-masks were used during fabrication. To reduce the fabrication costs and prevent hydrogen-based material from affecting the a-IGZO active layer during ES deposition [11], Uhm *et al.* proposed a two-photo-mask scheme that employed a gray-tone photomask to fabricate TFT devices [12]; however, the lack of an ES layer can cause damage to the a-IGZO active island when etching the S/D electrodes. In addition, the ZnO TFT with three photomasks was also proposed [13]. However, the continuous etching of S/D metal, IGZO and gate insulator were not considered in that report. The Table 1 lists the comparisons between these reports and this study.

**Table 1:** Summary of indium–gallium–zinc oxide (IGZO)-based thin-film transistors (TFTs) using different process.

Reference	Channel material	Self-aligned	Mask number	S/D Etching damage	a-IGZO Degradation due to ES-layer deposition
[10]	IGZO	Yes	4	No	Yes
[12]	IGZO	No	2	Yes	No
[13]	ZnO	Yes	3	No	No
this work	IGZO	Yes	2	No	No

## 2. Device Fabrication

**Figure 1:**
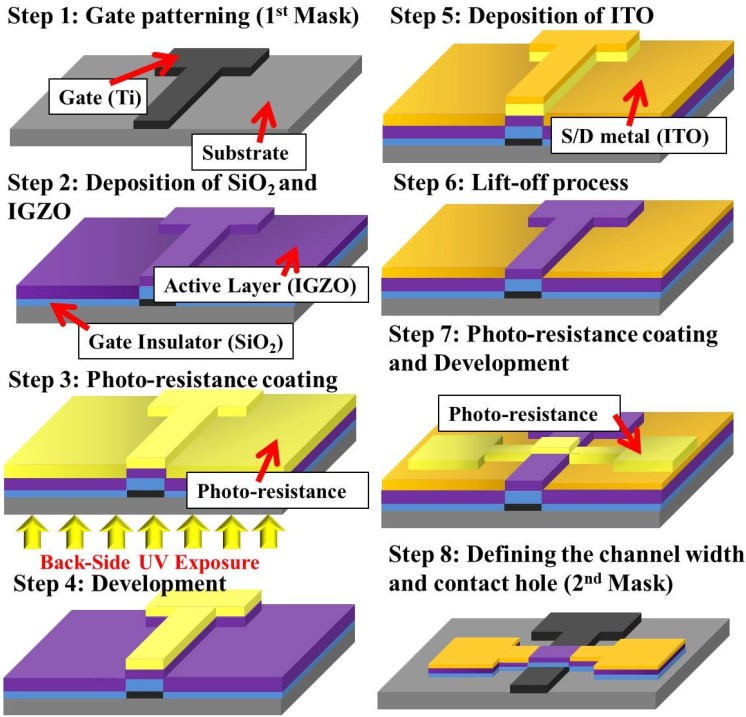
Two-Mask process flow of amorphous indium–gallium–zinc–oxide (a-IGZO) thin-film transistors (TFTs) with self-aligned structure.

Figure 1 shows the proposed two-mask process for fabricating a-IGZO TFTs. A 160-nm-thick Ti layer was first deposited onto a glass substrate by using thermal evaporation, and then patterned to form the gate electrode by the first photomask. Subsequently, a 200-nm-thick silicon dioxide (SiO_2_) was deposited using plasma enhanced chemical vapor deposition at 300 °C, forming the gate insulator. Subsequently, a 20-nm a-IGZO layer was deposited at 200 °C by a radio frequency (RF) sputtering system using a target of In:Ga:Zn = 1:1:1 in atomic ratio. The backside-lift-off (BLO) process is detailed as follows. First, a photo-resist (PR) was spin-coated onto IGZO and subjected to BUV exposure through the Ti gate as a photomask, as shown in Figure 1. Second, a 350-nm-thick Indium-Tin Oxide (ITO) was deposited using RF sputtering. Subsequently, the BLO scheme was applied to define the channel length of the self-aligned structure. Following the BLO process, the second photomask were used to define the channel width. Reactive-ion etching (RIE) with CF_4_ gas was used to continuously etch ITO, IGZO and SiO_2_ under the pressure of 80 mtorr. Finally, the devices were annealed at 200 °C for 30 min in a vacuum chamber. To compare the proposed devices, we used a traditional four-photo-mask process to fabricate devices that exhibited various overlap lengths between the gates and S/D electrodes [11].

## 3. Results and Discussion

In this study, a new two-photo-mask process with continuous-etching scheme was proposed for fabricating a-IGZO TFTs that exhibit self-aligned structures without ES layers. The ITO metal was designed as S/D metal to meet the requirement of the continuous etching process. Thus, S/D metal, IGZO and gate insulator can simultaneously be etched. Combining the BUV exposure and backside-lift-off (BLO) schemes can not only prevent the damage when etching the S/D electrodes but also reduce the number of photo-masks required during fabrication and minimize the parasitic capacitance at same time.

**Figure 2:**
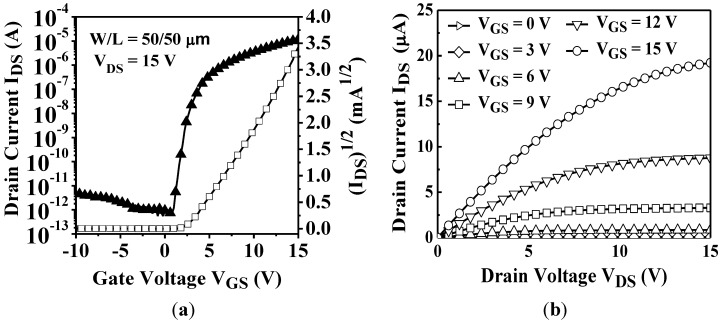
(**a**) Transfer characteristic (*V*_DS_ = 15 V) and (**b**) output characteristic of a-IGZO TFT with self-aligned structure (*W*/*L* = 50 μm/50 μm).

Figure 2a,b shows the transfer and output characteristics of the proposed a-IGZO TFTs with self-aligned structure for channel width (W) of 50 μm and channel length (L) of 50 μm, respectively. A total of 20 devices were measured at various positions across the substrate by using a semiconductor parameter analyzer (HP4145B Hewlett Packard, Palo Alto, CA, USA). The extracted saturation field-effect mobility (μ_sat_), threshold voltage (*V*_T_), subthreshold swing (*S*), and on-off current ratio (*I*_on_/*I*_off_) are 9.50 cm^2^/V·s, 3.39 V, 0.3 V/decade, and 4 × 10^7^, respectively. μ_sat_ and *V*_T_ are extracted from the slope and linear extrapolations of the plot of the square root of the drain current as a function of the gate voltage, respectively [14,15,16]. Moreover, the output characteristics show that current crowding did not occur in the linear region to reveal the contact resistance of the S/D regions can satisfy the requirement of transportation current [17].

**Figure 3:**
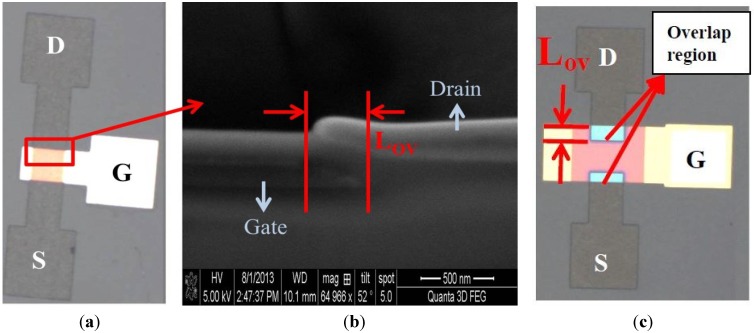
(**a**) Optical microscopy images of a-IGZO TFTs with self-aligned structures; (**b**) Scanning electron microscopy images of a-IGZO TFTs with self-aligned structures; (**c**) Optical microscopy images of a-IGZO TFTs with overlap structures.

The devices were fabricated using the traditional four-photo-mask process, yielding various overlap lengths (*L*_ov_ = 100 and 200 μm) between the gate and S/D electrodes. Figure 3 shows optical microscopy images and scanning electron microscopy images of the a-IGZO TFTs, which exhibit self-aligned and overlapping structures. In Figure 3a,b, the overlapping region less than 0.5 μm could be observed in scanning electron microscopy images. The overlapping region was clearly observable among the devices fabricated using the traditional four-photo-mask process in Figure 3c, which yielded parasitic capacitance between S/D and gate electrodes (*C*_gd_, *C*_gs_). The parasitic capacitance was proportional to the area of overlapping region (*W* × *L*_ov_), yielding increased feed-through voltage, noise, and circuit delay in devices typically used in displays applications [12].

**Figure 4:**
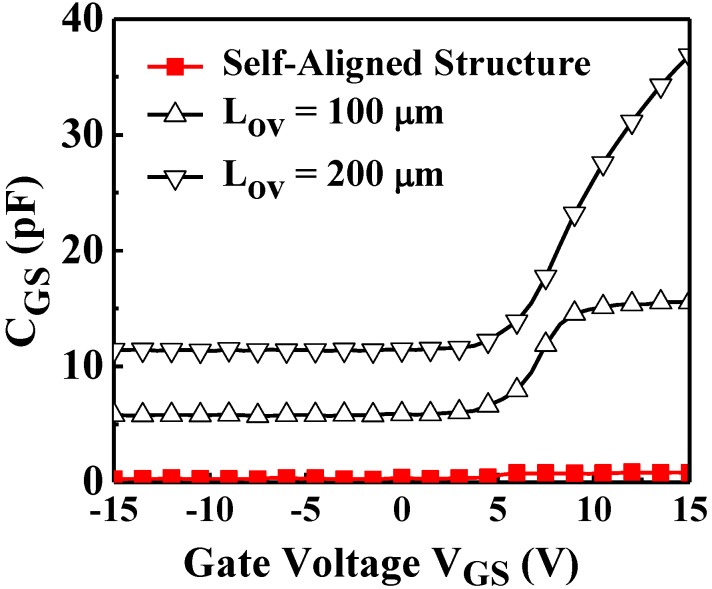
Gate-to-source capacitance (*C*_gs_) of a-IGZO TFTs with self-aligned structure and overlap structure for different *L*_ov_ (*W* = 200 μm).

**Figure 5:**
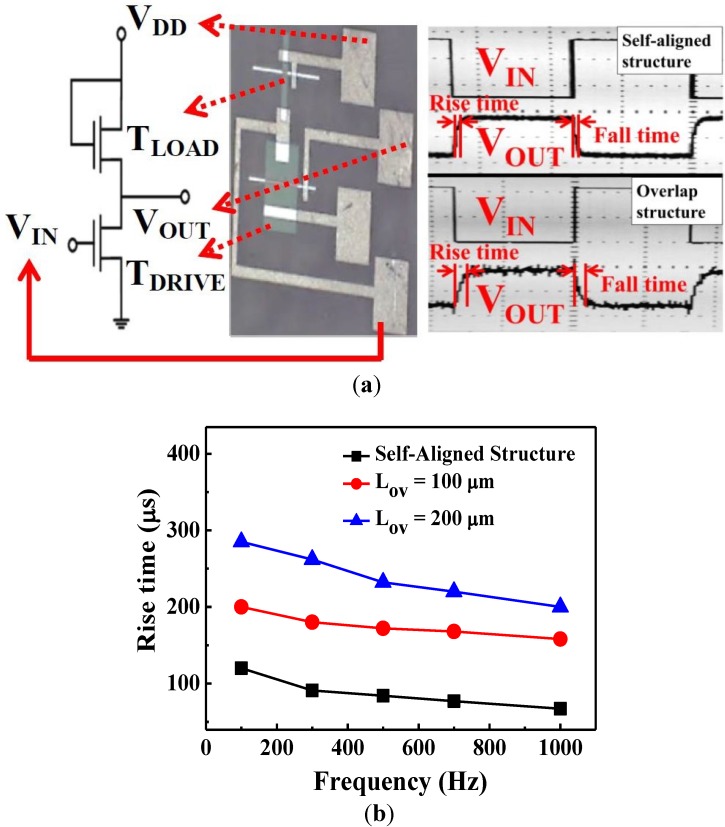
(**a**) Inverter composed of the fabricated a-IGZO TFTs (*V*_DD_ = 20 V, *W*_Drive_ = 200 μm, *L*_Drive_ = 10 μm, *W*_Load_ = 40 μm, and *L*_Load_ = 10 μm); (**b**) The extracted rise time for inverters composed of a-IGZO TFTs with self-aligned structure and overlap structure for *L*_ov_ = 100 and 200 μm.

To analyze the parasitic capacitance between the S/D and gate electrodes, the capacitance–voltage of the fabricated a-IGZO TFTs was measured using an Inductance-Resistance-Capacitance (LCR) meter (HP4284A, Hewlett Packard, Palo Alto, CA, USA; Figure 4). The capacitance markedly increased in conjunction with the overlap length (*L*_ov_) (*i.e.*, the area of overlapping region). The device that was fabricated using the traditional four-photo-mask process (*i.e.*, *L*_ov_ of 200 μm) exhibited a minimal capacitance of 11.46 pF, which was considerably higher compared with that of the device fabricated using the proposed method (0.25 pF). Hence, the self-aligned structure reduced the parasitical capacitance between the S/D and gate electrodes. To examine the effects of parasitic capacitance, two types of the inverters were fabricated (Figure 5a); the first comprised the proposed two-mask process with self-aligned structures, and the second comprised overlapping structures (both types employed an N-type metal-oxide-semiconductor configuration as the active load). Figure 5a also shows the dynamic state measurements for the inverters. Various square wave frequencies (*V*_IN_) were input into the inverters, and the output signals (*V*_OUT_) were measured using oscilloscope. The rise time of the output signal was defined as the time required by the signal to rise from 10% to 90% of the step height; this time is related to the parasitic capacitance of TFTs due to the Resistance-Capacitance (RC) delay for inverter circuit operation. Figure 5b indicates that the rise time (delay time) increased in conjunction with the overlap length (*L*_ov_), (*i.e.*, the parasitic capacitances). Compared with inverter with *L*_ov_ of 200 μm, the inverter that comprised the proposed two-mask schemes with self-aligned structure exhibited a substantial decrease in delay time from 230 to 78 μs (the input frequency is 500 Hz).

## 4. Conclusions

In this study, a novel two-photo-mask process was proposed for fabricating a-IGZO TFTs that exhibit a self-aligned structure. Combining the BUV exposure and BLO schemes reduced both the parasitic capacitance and number of photo-masks required for fabrication. In addition, the proposed devices that lack ES layers yield an undamaged a-IGZO active layer, facilitating superior performance levels (such as a field-effect mobility of 9.5 cm^2^/V·s, a threshold voltage of 3.39 V, and a subthreshold swing of 0.3 V/decade). Compared with using a traditional four-photo-mask process and no ES layer, the proposed process can be used to fabricate a-IGZO TFTs that exhibit fair performance levels. Moreover, the reduced parasitic capacitance yielded a marked decrease in delay time in an inverter fabricated using the proposed method. Thus, the proposed process is a suitable candidate for use in a-IGZO TFT applications, such as active-matrix organic light-emitting diodes (AMOLED), because it achieves high operational speeds and reduces fabrication costs.
